# Soil pedostructure-based method for calculating the soil-water holding properties

**DOI:** 10.1016/j.mex.2018.08.006

**Published:** 2018-08-18

**Authors:** Amjad T. Assi, Rabi H. Mohtar, Erik Braudeau

**Affiliations:** a Department of Biological and Agricultural Engineering, Texas A&M University, College Station, TX 77843-2117, USA; b Zachry Department of Civil Engineering, Texas A&M University, College Station, TX 77843-2117, USA; c Faculty of Agricultural and Food Sciences, American University of Beirut, Beirut 1107 2020, Lebanon

**Keywords:** Pedostructure method for calculating the field capacity, Permanent wilting point, Available water, Soil aggregates structure, Thermodynamic, Field capacity, Permanent wilting point, Available water

## Abstract

Soil aggregates structure (pedostructure) plays a pivotal role in regulating water and nutrient circulation, and consequently defines soil health, productivity, and water use efficiency. However, the soil aggregates structure is not currently considered in the quantification of soil-water holding properties. The authors applied a thermodynamic and soil structure-based approach to quantify soil-water holding properties. The paper provides a methodology, based on pedostructure concept, to quantify field capacity (FC), permanent wilting point (PWP), and available water (AW). The validity of the developed method was tested through application to two types of soil: a loamy fine sand soil and a silt loam soil. The calculated values for FC, PWP, and AW were compared with the FAO recommended values of FC, PWP and AW. For the loamy fine sand, the calculated values were: FC = 0.208 m^3^/m^3^, PWP = 0.068 m^3^/m^3^, and AW = 0.140 m^3^/m^3^ all of which fall within the recommended values of FAO for such a soil type. Similarly, the calculated values for the silt loam were: FC = 0.283 m^3^/m^3^, PWP = 0.184 m^3^/m^3^, and AW = 0.071 m^3^/m^3^ all were in agreement with the FAO recommended ranges for such a soil type.

• A thermodynamic, structure-based approach for soil water holding properties.
• Unique solutions for quantifying both field capacity and permanent wilting point.

## Graphical abstract

**Figure:**
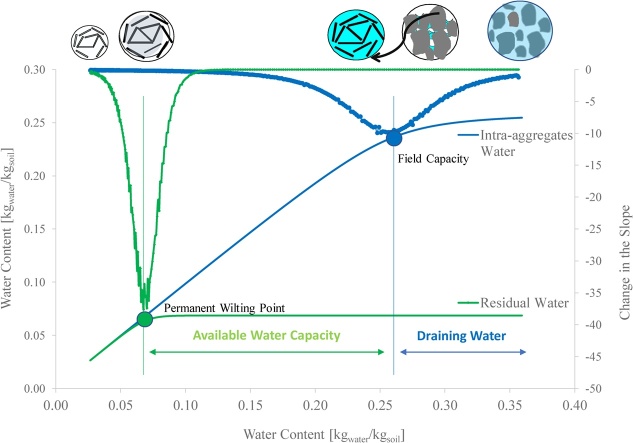

**Specifications Table**

**Table:** 

Subject area	*Agricultural and Biological Sciences*
More specific subject area	*Soil-Water Holding Properties*
Method name	*Pedostructure Method for Calculating the Field Capacity, Permanent Wilting Point, and Available Water*
Name and reference of original method	*New method.*
Resource availability	https://wefnexus.tamu.edu/hydrostructural-pedology/

## Method details

**Fig. 1:**
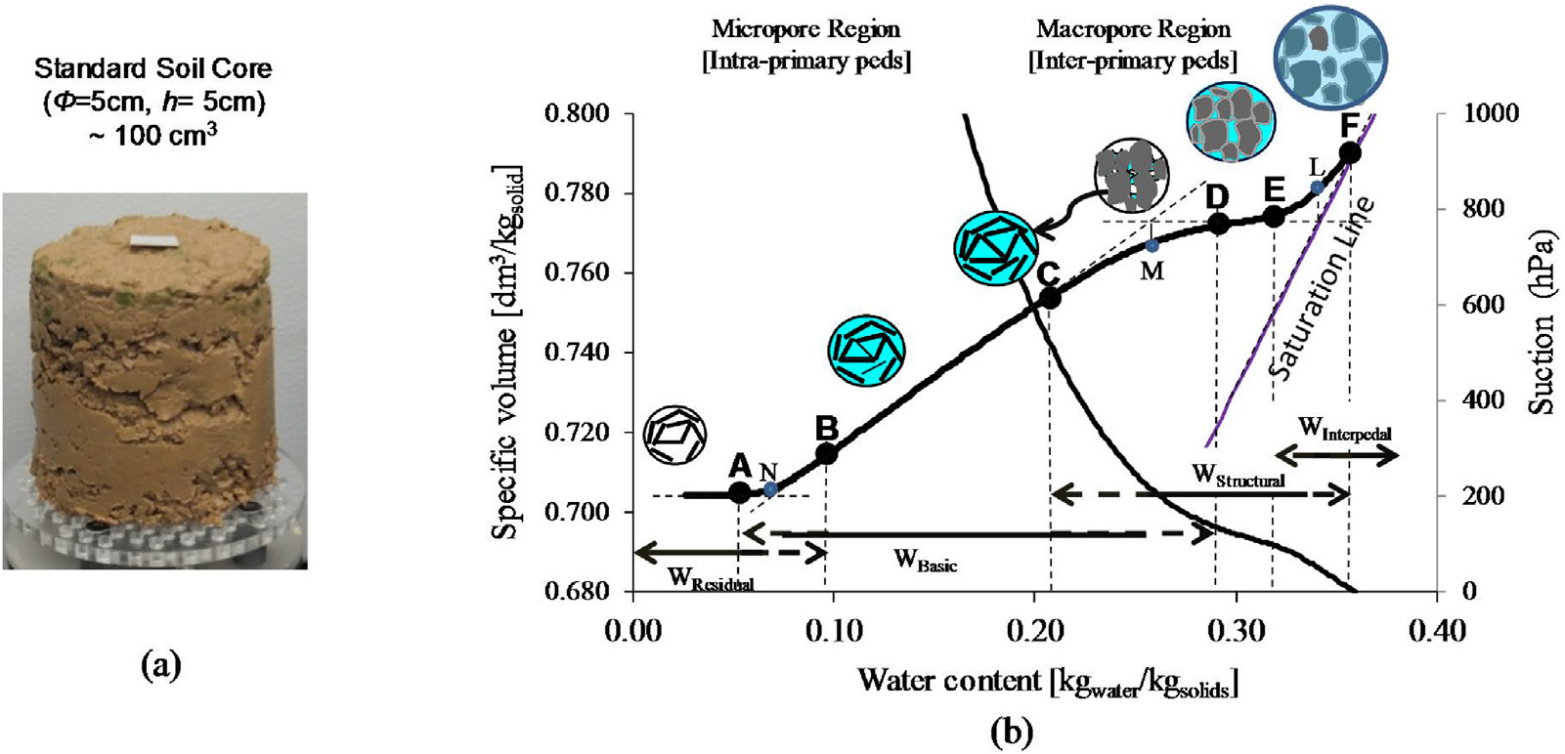
Pedostructure concept: (a) a standard soil core to represent the pedostructure of a soil horizon, (b) delineating the two water types of a pedostructure by soil shrinkage curve (ShC) and water retention curve. On the ShC, points (A, N, B, C, M, D, E, L, and F) are the characteristic points of the water pools of the different shrinkage phases: interpedal, structural, basic and residual.

**Fig. 2:**
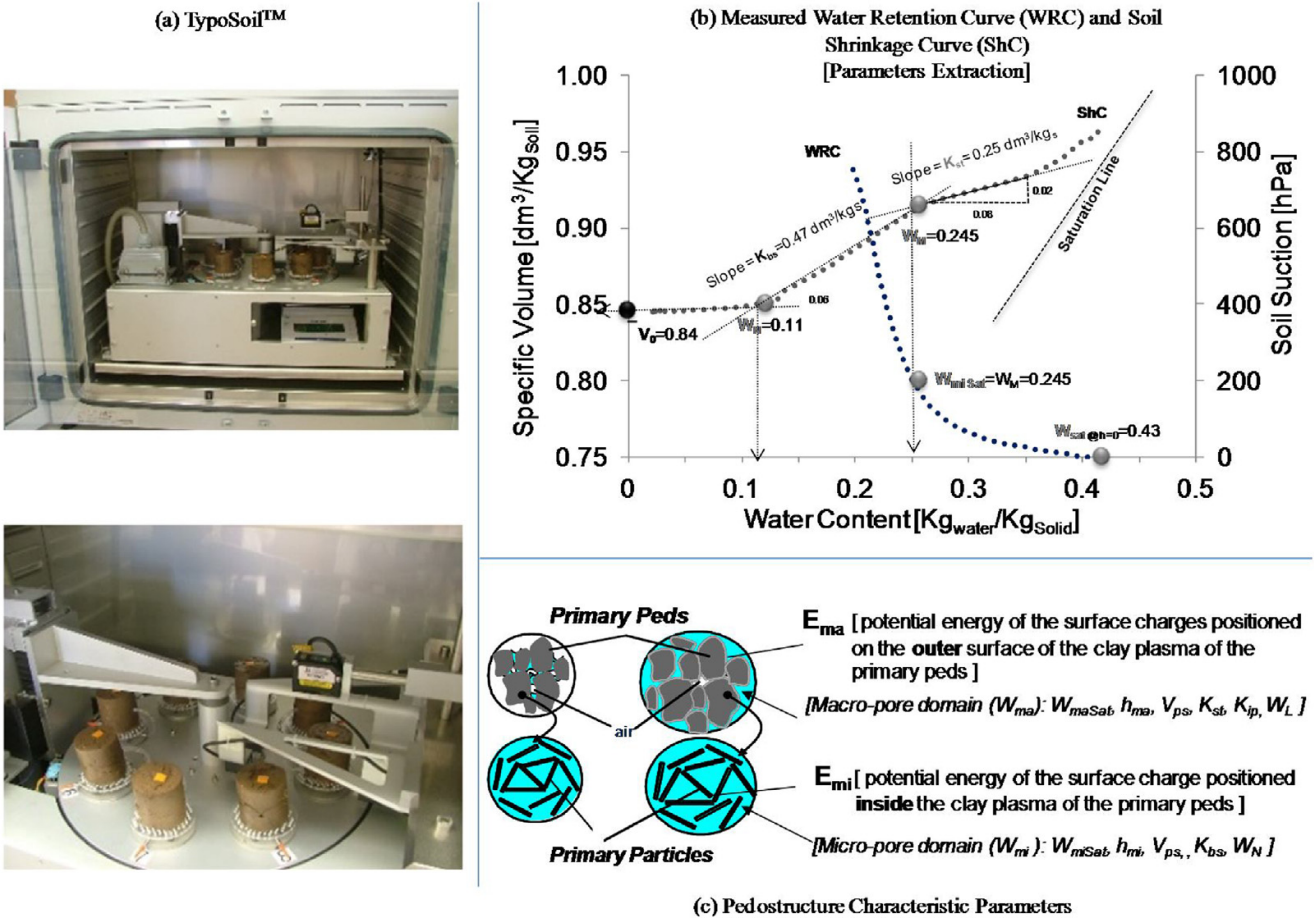
Hydrostructural characterization of pedostructure. (a) TypoSoil^™^ apparatus, (b) identifying the characteristic parameters on the measured water retention curve and soil shrinkage curve of a pedostructure; (c) representation of the characteristic parameters of a pedostructure.

This work introduces a new methodology for calculating the field capacity (FC), Permanent Wilting Point (PWP), and available (AW), using the soil aggregates structure (pedostructure) instead of soil texture. The Pedostructure approach was developed from the pedological description of the level of soil aggregates organization in which the primary particles (minerals “sand, silt, clay” and natural organic matter) assemble to form “primary” peds. These primary peds then aggregate to form the pedostructure (soil aggregates structure) as described by Braudeau et al. [1]. Pedostructure can be practically taken using a standard soil core (Fig. 1a) to represent the unique soil organization of the horizon from which it was taken. Each soil type has a unique pedostructure whose hydro-structural properties (pore systems and potential energies of surface charges on the primary peds) can be described using various hydrostructural parameters [2]. These parameters are extracted from the continuously and simultaneously measured data points of the water retention curve (WRC: the curve of soil water content vs. soil suction) and soil shrinkage curve (ShC: the relationship between the soil water content and the soil volume) produced by the TypoSoil^™^ device (Fig. 2a).

**Fig. 3:**
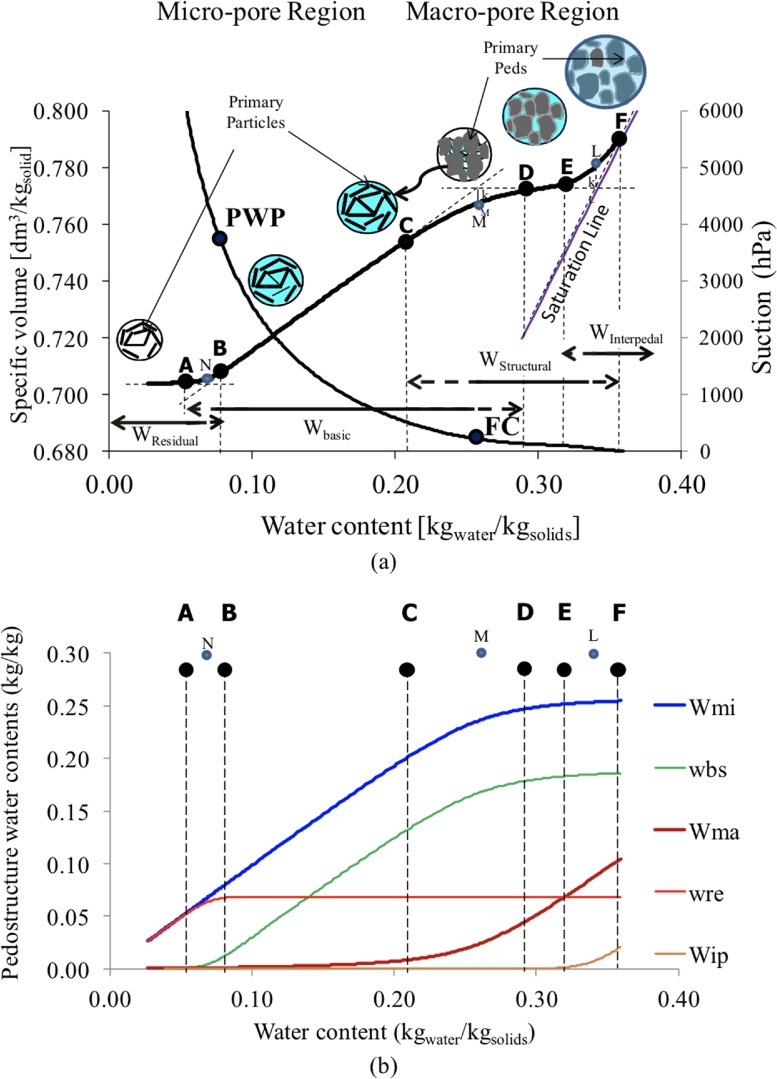
Pedostructure water contents: (a) characteristic points of the pedostructure water contents and the corresponding soil-water holding properties; (b) modeling the pedostructure water contents from saturation to dry state. This thermodynamic and structure-based modeling identifies the efficient contribution of the different water pore systems as a respond of soil-water loss, and thus it can be used to identify the water-holding characteristic properties of a specific soil type and soil horizon.

According to the thermodynamic formulation of the WRC and the ShC [2], one should recognize that there are two pore regions within a pedostructure (Fig. 2b):

1 **Micro-pore region,** representing the pore volume and structure *inside* the primary peds. Its water content is called micro-water content ($W_{mi}$). The following characteristic points of micro-pore water content are unique for each soil type (Fig. 1b): $W_{M}$ is the water content equivalent to the micro-pore volume at saturation; $W_{C}$ is the water content at the beginning of the structural shrinkage curve; $W_{B}$ is the water content at the air entry point of micro-pore structure; $W_{N}$ is the water content equivalent to the minimum micro-pore volume, or the saturated residual water content ${(w}_{reSat})$; and $W_{A}$ is the water content at the shrinkage limit.
2 **Macro-pore region**, representing the pore volume and structure *outside* the primary peds. It may contain two types of water: (a) non-swelling macro-water, known as macro-water content ($W_{ma}$), and (b) swelling macro-water or interpedal water content ($W_{ip}$), which corresponds to the interpedal saturation shrinkage phase of the shrinkage curve. This shrinkage phase has a slope of 1 ($K_{ip}=1)$, and its presence only occurs when the inter-primary peds porosity is saturated with water and the soil has the ability to hold more water by spacing the aggregates: hence causing the sample to swell. Thus, the soil water content is $W^{'}=\;W_{mi}+\;W_{ma}+W_{ip}$, or in the case where there is no interpedal water, the soil water content is $W=\;W_{mi}+\;W_{ma}$. The following water-holding characteristic points of macro-pore water content are unique for each soil type (Fig. 3): $W_{D}$ is the water content at the beginning of the effective shrinkage of the primary aggregates. In which, $W_{D}$ represents the water content at which the micro-pore water content begins to contribute the lost water from the macro-pore system. $W_{E}$, $W_{L}$, $W_{F}$ are the characteristic points of the interpedal water content $\left(W_{ip}\right)$, at the lower limit of the interpedal shrinkage phase, the total non-swelling water content of pedostructure, and the total water content at saturation, respectively. In case there is no interpedal water, then there will be no (E) point, and $W_{L}$ will represent the saturated water content ($W_{Sat}$).

## Materials and methods

This work builds on the work of Assi et al. [3] and Braudeau et al. [2] to develop a methodology for calculating the FC, PWP, and AW.

### Part 1: soil samples collection, preparation, and characterization

Note: the procedures mentioned hereafter have been well described in previous peer-reviewed papers [2,3], a book [4], and a user manual [5]. Spreadsheets are available to treat the data obtained from the TypoSoil^™^ and will be provided as supplementary material to this paper. This section presents the steps to be done and references the published work for more details.

1 *Collect the soil samples*. Standard cylindrical stainless-steel soil cores (Φ = 5 cm, h = 5 cm) are used to collect the soil samples from the field. Each soil sample represents the pedostructure of the soil horizon from which it was taken. In this study, two soil types were sampled, and soil samples were taken from the top horizon. The soil types are: (1) Chazos **loamy fine sand** soil, an Alfisol, from College Station, TX, USA, with a texture of: 4% clay, 13% silt, and 83% sand; and (2) Sabkha **silt loam** soil, an Aridisol, from Al Khor, Qatar, with a texture of: 15% clay, 65% silt, and 20% sand.
2 *Prepare the soil samples for TypoSoil*^™^ *measurement* (Fig. 2a). TypoSoil^™^ [6] provides continuous and simultaneous measurement of three state variables for 8 soil samples in each run (Table 1): moisture content (measured by a balance “MASSE”), soil suction (measured by ceramic cup tensiometers “TENSIO”), and specific volume (measured by two laser beams “BARR 1 and BARR 2”, and 1 laser spot “SPOT”). These state variables will be used to construct the WRC and ShC. The preparation procedures are outlined in the TypoSoil^™^ user manual [5]. Note: in case a user has measured the WRC and the ShC by other apparatus than TypoSoil, she will still be able to use the spreadsheets but the user needs to go to step 4 directly. In step 4, (a) for WRC: a user can insert the measured water content (gravimetric water content - W) and the corresponding soil suction (h measure) in hPa (cm); and (b) for ShC: a user can insert the measured water content (gravimetric water content - W) and the corresponding specific volume (dm^3^/kg_soil_) (vcalc).

**Table 1:** TypoSoil^™^ raw data.

Slot	2					
Code	RR LOC2 UD					
Date	25/7/2013 – 8 h 31					
Batch	TYPO130725					
Operator	Josh					
GenDate	20130728 - 093755					
“Entetes”	“TEMPS”	“BARR1”	“BARR2”	“SPOT”	“MASSE”	“TENSIO”
Data	79	0	856	1156	187.54	1934
Data	658	709	198	1185	187.29	1931
Data	1227	708	202	1183	187.13	1930
3 *Construct the water retention curve (WRC) and the soil shrinkage curve (ShC)*. To construct the WRC and the ShC, the soil water content and the soil specific volume need to be calculated from the measured state variables, such that:

(1) $$W=\frac{\left(m-M_{s}\right)}{M_{s}}$$

where, W is the water content of the soil sample $\left[{\text{k}\text{g}}_{\text{water}}\;{\text{kg}}_{\text{solid}}^{\text{-1}}\right]$, m is the measured mass of the soil sample ${\text{[kg}}_{\text{water}}]$, $M_{s}$ is the dry mass of the soil sample at 105 °C ${\text{[kg}}_{\text{solid}}]$.

(2) $$\bar{V}=\frac{\pi D^{2}H}{4M_{s}}\times 10^{-4}$$

where, $\bar{V}$ is the specific volume of the soil sample $\left[{\text{dm}}^{3}\;{\text{kg}}_{\text{solid}}^{\text{-1}}\right]$, D and H are, respectively, the measured diameter and height of the soil sample by the laser sensors [dm], $M_{s}$ is the dry mass of the soil sample at 105 °C ${\text{[kg}}_{\text{solid}}]$.

- The water retention curve (WRC) is constructed by drawing the calculated soil water content (W $\left[{\text{kg}}_{\text{water}}\;{\text{kg}}_{\text{solid}}^{\text{-1}}\right]$) vs. the measured soil suction (h [dm ∼ kPa]).
- The soil shrinkage curve (ShC) is constructed by drawing the calculated soil water content (W $\left[{\text{kg}}_{\text{water}}\;{\text{kg}}_{\text{solid}}^{\text{-1}}\right]$) vs. the calculated specific volume $\bar{V}$ $\left[{\text{dm}}^{3}\;{\text{Kg}}_{\text{solid}}^{\text{-1}}\right]$.
- *Extract the characteristic parameters of the pedostructure* (Fig. 2b, c) by adjusting the thermodynamic equations of WRC and ShC [2] with the measured ones by TypoSoil^™^. Again, the procedures of extracting these parameters were explained in Assi et al. [3] and chapter 6 of Braudeau et al., [4].
- *Model the pedostructure water contents* (Fig. 3). The spreadsheets will then use these extracted parameters to calculate the different pedostructure water contents: interpedal water content ($W_{ip}$) (Eq. 3); macro-pore water content ($W_{ma}$) (Eq. 4), micro-pore water content ($W_{mi}$) (Eq. 5), basic water content ($w_{bs}$) (Eq. 6), and residual water content ($w_{re}$) (Eq. 7). These values can be calculated by using the following equations. Table 2 below provides a summary of the state variables, pedostructure water contents and the needed parameters to calculate each of them.

**Table 2:** The state variables and the corresponding parameters of the pedostructure WRC and ShC.

Symbol	Definition	Unit	Corresponding hydro-structural parameters
$W_{Sat}$	Pedostructure saturated water content	${\text{kg}}_{\text{water}}\;{\text{kg}}_{\text{soil}}^{\text{-1}}$	$W_{maSat}^{eq}$, $W_{miSat}^{eq}$
W	Pedostructure water content	${\text{kg}}_{\text{water}}\;{\text{kg}}_{\text{soil}}^{\text{-1}}$	$\bar{E}/A$, ${\bar{E}}_{ma}/A$
$W_{mi}^{eq}$	Micropore water content of the pedostructure	${\text{kg}}_{\text{water}}\;{\text{kg}}_{\text{soil}}^{\text{-1}}$	
$W_{ma}^{eq}$	Macropore water content of the pedostructure	${\text{kg}}_{\text{water}}\;{\text{kg}}_{\text{soil}}^{\text{-1}}$	
$h^{eq}\left(W\right)$ $h_{mi\;}{(W}_{mi}^{eq})$ $h_{ma\;}{(W}_{ma}^{eq})$	Pedostructure water potential which is in instantaneous equilibrium between inside and outside the primary peds, such that: $h_{mi}=h_{ma}=h$	dm ∼ kPa	$W_{maSat}^{eq},$$W_{miSat}^{eq}$ ${\bar{E}}_{ma}$, ${\bar{E}}_{mi}$
$\bar{V}$	the specific volume of the pedostructure	${\text{dm}}^{3}\;{\text{kg}}_{\text{soil}}^{\text{-1}}$	${\bar{V}}_{o}$,$\;K_{bs}$,$\;K_{st}$, $K_{ip}$
$w_{re}^{eq}$	Specific water content of the water pool associated to the residual linear shrinkage phase of the pedostructure	${\text{kg}}_{\text{water}}\;{\text{kg}}_{\text{soil}}^{\text{-1}}$	$k_{N}$, $W_{N}$
$w_{bs}^{eq}$	Specific water content of the water pool associated to the basic linear shrinkage phase of the pedostructure	${\text{kg}}_{\text{water}}\;{\text{kg}}_{\text{soil}}^{\text{-1}}$	
$w_{st}^{eq}$	Specific water content of the water pool associated to the structural linear shrinkage phase of the pedostructure	${\text{kg}}_{\text{water}}\;{\text{kg}}_{\text{soil}}^{\text{-1}}$	
$w_{ip}^{eq}$	Specific water content of the water pool associated to the interpedal linear shrinkage phase of the pedostructure, parallel to the saturation line	${\text{kg}}_{\text{water}}\;{\text{kg}}_{\text{soil}}^{\text{-1}}$	$k_{L}$, $W_{L}$

(3) $$w_{ip}=\frac{1}{k_{L}}\ln\left[1+exp\left(k_{L}\left(W-W_{L}\right)\right)\right]$$

(4) $$W_{ma}^{eq}(W)=\frac{\left(W+\frac{\bar{E}}{A}\right)+\sqrt{\left[{\left(W+\frac{\bar{E}}{A}\right)}^{2}-\left(4\frac{{\bar{E}}_{ma\;}}{A}W\right)\right]}}{2}$$

(5) $$W_{mi}^{eq}(W)=W-W_{ma}^{eq}=\frac{\left(W-\frac{\bar{E}}{A}\right)-\sqrt{\left[{\left(W+\frac{\bar{E}}{A}\right)}^{2}-\left(4\frac{{\bar{E}}_{ma\;}}{A}W\right)\right]}}{2}$$

(6) $$w_{bs}=\frac{1}{k_{N}}\ln\left[1+exp\left(k_{N}\left(W_{mi}^{eq}-W_{miN}^{eq}\right)\right)\right]$$

(7) $$w_{re}=W-\frac{1}{k_{N}}\text{ln}\left[1+exp\left(k_{N}\left(W-W_{miN}^{eq}\right)\right)\right]$$

Where,

W pedostructure water content excluding the saturated interpedal water $\left[{\text{kg}}_{\text{water}}\;{\text{kg}}_{\text{soil}}^{\text{-1}}\right]$,

$W_{ma}$ gravimetric macro-pore water content “outside the primary peds” $\left[{\text{kg}}_{\text{water}}\;{\text{kg}}_{\text{soil}}^{\text{-1}}\right]$,

$W_{mi}$ gravimetric micropore water content “inside the primary peds” $\left[{\text{kg}}_{\text{water}}\;{\text{kg}}_{\text{soil}}^{\text{-1}}\right]$,

${\bar{E}}_{ma\;}$ potential energy of surface charges positioned on the outer surface of the clay plasma of the primary peds $\left[\text{J}\;{\text{kg}}_{\text{solid}}^{\text{-1}}\right]$,

${\bar{E}}_{mi\;}$ potential energy of surface charges positioned inside the clay plasma of the primary peds $\left[\text{J}\;{\text{kg}}_{\text{solid}}^{\text{-1}}\right]$,

$k_{N}$ and $k_{L}$ represent the vertical distance between the intersection points of the two tangents at points N, and L (Fig. 1b) and the measured shrinkage curve, respectively $\left[{\text{kg}}_{\text{soil}}\;{\text{kg}}_{\text{water}}^{\text{-1}}\right]$,

$W_{miN}^{eq}$ micro-pore water content calculated by (Eq. 5) but by using $W_{N}$ instead of W,

$W_{N}$ water content at the intersection point (N) in (Fig. 1b) and represents the water content of the primary peds at dry state such that $W_{N}=\text{max}(w_{re})$ $\left[{\text{kg}}_{\text{water}}\;{\text{kg}}_{\text{soil}}^{\text{-1}}\right]$,

$w_{re}$ water pool associated with the residual shrinkage phase of the shrinkage curve $\left[{\text{kg}}_{\text{water}}\;{\text{kg}}_{\text{soil}}^{\text{-1}}\right]$,

$W_{L}$ water content at the intersection point (L) (Fig. 1b) such that $W_{L}=W_{M}+\text{max}(w_{st})$ $\left[{\text{kg}}_{\text{water}}\;{\text{kg}}_{\text{soil}}^{\text{-1}}\right]$,

$W_{M}$ water content at the intersection point (M) (Fig. 1b) such that $W_{M}=W_{N}+\text{max}(w_{bs})$ and it represents the saturated water content of the micropore domain $\left[{\text{kg}}_{\text{water}}\;{\text{kg}}_{\text{soil}}^{\text{-1}}\right]$.

### Part 2: define and calculate the field capacity (FC), permanent wilting point (PWP), and available water (AW) based on the pedostructure water contents

**Fig. 4:**
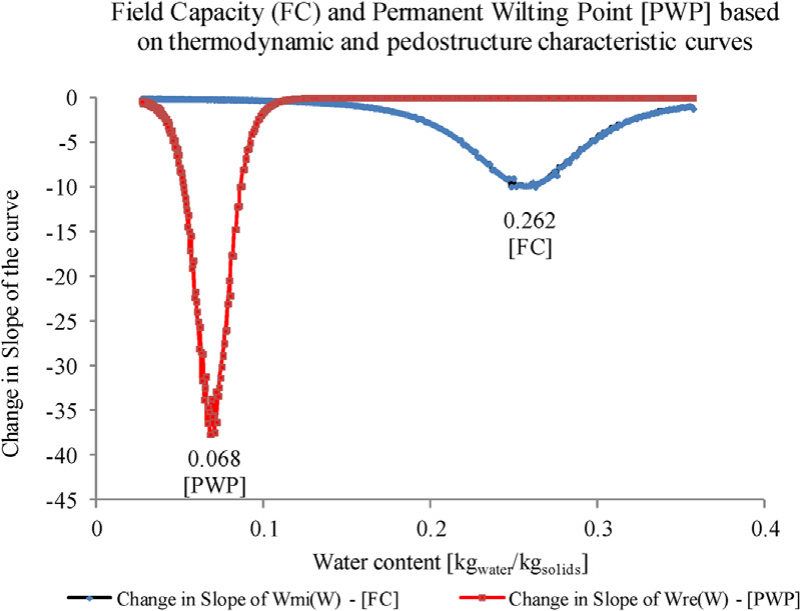
Identify the value of field capacity based on the maximum change of the slopes in pedostructure micro-pore water curve and the permanent wilting point based on the maximum change of the slope in the pedostructure residual water curve.
• **Permanent Wilting Point (**${\bar{W}}_{PWP}$**)**: the water content at PWP corresponds to the water content at the air entry point of micro-pore domain. At this point, a capillary break within the micro-porosity of primary peds occurs and the water cannot be reached by the plant roots at the contact surface of the peds [9]. This water content corresponds to point B in Fig. 3. At this point, the soil suction is around pF4.2 (i.e. soil suction: 3791 hPa which is equivalent to 15,000 hPa air pressure as applied in Richards' apparatus). So, Point B can represent the maximum changes in the slope of the residual water content curve [w_re_ (W)], as shown in Fig. 3, the soil water content at this maximum change in slope will be used as permanent wilting point.
• **Available Water Capacity (**AW***)***: available water capacity can be then identified as the difference between the FC and PWP, such that:

The authors made use of the modelled pedostructure water contents to calculate the field capacity (FC), permanent wilting point (PWP), and available water (AW). These values can be calculated such that:

• **Field capacity (**${\bar{W}}_{FC}$**)**: the water content at field capacity corresponds to the water content at which the thermodynamic forces between soil and water are much higher than the gravitational forces to appoint where the water flux out of soil medium is negligible. Based on the thermodynamic understanding of pedostructure, as explained earlier, this water content can then be identified by the rapid change in the micro-pore water content curve. Therefore, FC of a soil occurs at the maximum of the change in slope of the $W_{mi}$ curve. This value can be identified by finding the root of the third derivative of $W_{mi}$ curve, or by numerical solutions (Fig. 4). At this point, all the interpedal water will have vanished.

(8) $$AW={\bar{W}}_{FC}-{\bar{W}}_{PWP}$$

• **Unit Conversion**: The calculated soil water holding properties: field capacity, permanent wilting point, and available water are calculated as gravimetric water contents (${\text{kg}}_{\text{water}}\;{\text{kg}}_{\text{soil}}^{\text{-1}}$). To be able to compare the results with existing methods, the gravimetric water contents as reported in pedostructure method (${\text{kg}}_{\text{water}}\;{\text{kg}}_{\text{soil}}^{\text{-1}}$) need to be converted to volumetric water contents (${\text{m}}^{3}\;{\text{m}}^{\text{-3}}$). The conversion from gravimetric water content into volumetric water content was done as following:

(9) $$\theta_{FC}={\bar{W}}_{FC}\left(\frac{\rho_{FC}}{\rho_{w}}\right)$$

(10) $$\theta_{PWP}={\bar{W}}_{PWP}\left(\frac{\rho_{PWP}}{\rho_{w}}\right)$$

where, $\theta_{FC}$ and $\theta_{PWP}$ are the volumetric water contents at field capacity and permanent wilting point, respectively [m^3^_water_/m^3^_soil_], $\rho_{FC}$ and $\rho_{PWP}$ are the bulk densities of the soil at field capacity and permanent wilting point, respectively [kg_soil_/m^3^_soil_], and $\rho_{w}$ is the specific density of water [kg_water_/ m^3^_water_]. Where $\rho_{FC}=\;1/{\bar{V}}_{FC}$, and $\rho_{PWP}=\;1/{\bar{V}}_{PWP}$. Here, ${\bar{V}}_{FC}$ and ${\bar{V}}_{PWP}$ are the specific volumes at the field capacity and permanent wilting point as observed in the soil shrinkage curve, respectively.

## Additional information

There are different approaches and recommended values to estimate the field capacities and permanent wilting points. There are variations in the recommended values, even by the most standard method. These soil-water holding properties are highly affected by the soil structure and soil organic matter. FAO estimation [7] and the Department of Agriculture Bulletin 462 are among the standard and widely used methods for estimating the FC, PWP, and AW values. However, one can observe some noticeable variations between their estimation “recommended” values based on the soil texture. For example, for a silty clay loam soil, FAO suggests a field capacity in a range of 0.300–0.370 m^3^/m^3^, whereas, Bulletin 462 suggests an average value of 0.28 m^3^/m^3^. One can recognize that the suggested value by Bulletin 462 [8] is outside of the recommend range of values by FAO.

In this paper, the focus was on building a standard methodology for estimating field capacity and permanent wilting point that consider the soil aggregates structure. As shown in Table 3, most of the calculated values for FC and PWP were in good agreement with the recommended values by FAO.

**Table 3:** The estimated values of the field capacity (FC), permanent welting point (PWP) and available water (AW) based on the pedostructure method and the corresponded range of values as recommended by FAO [7].

Soil Sample	Pedostructure Method					FAO Method		
	${\bar{W}}_{FC}$ (Kg/kg)	$\theta_{FC}$ (m^3^/m^3^)	${\bar{W}}_{PWP}$ (Kg/kg)	$\theta_{PWP}$ (m^3^/m^3^)	**AW** (m^3^/m^3^)	$\theta_{FC}$ (m^3^/m^3^)	$\theta_{PWP}$ (m^3^/m^3^)	**AW** (m^3^/m^3^)
Chazos Soil [Loamy fine sand]	0.144	0.208	0.047	0.068	0.140	0.110–0.190	0.030–0.100	0.010 – 0.160
Sabkha Soil [Silt loam]	0.247	0.283	0.212	0.184	0.071	0.220–0.360	0.090–0.210	0.010 – 0.270
