# Supplementary material for: Soil pedostructure-based method for calculating the soil-water holding properties
Source: MethodsX. 2018 Aug 18;5:950–8. doi: 10.1016/j.mex.2018.08.006 (PMC6111243; doi:10.1016/j.mex.2018.08.006)
Supplement: Supplementary file 1 [file mmc1.docx]

**Supplementary material *and/or* Additional information:**

Attached: Pedostructure Method – MethodX spreadsheets:

- *Sheet 1*: **TypoSoil Data** – the raw data as measured by TypoSoil^TM^.
- *Sheet 2*: **Mes.WRC & ShC** – convert the TypoSoil^TM^ data to three state variables “water content, soil suction, and specific volume” to construct the two soil-water characteristic curves: water retention curve (WRC), and soil shrinkage curve (ShC).
- *Sheet 3*: **Mod. WRC** – extract the hydro-structural parameters of the water retention curve (WRC).
- *Sheet 4*: **Mod. ShC** – extract the hydro-structural parameters of the soil shrinkage curve (ShC), and modeling the different pedostructure water contents: interpedal water content ($W_{ip}$); macro-pore water content ($W_{ma}$), micro-pore water content ($W_{mi}$), basic water content ($w_{bs}$), and residual water content ($w_{re}$).
- *Sheet 5:* **FC &PWP** – calculate the field capacity, permanent wilting point, and available water.
